# Designing the Diagnostic Criteria for Disseminated Intravascular Coagulation (DIC)

**DOI:** 10.14789/jmj.JMJ23-0038-P

**Published:** 2023-12-20

**Authors:** JECKO THACHIL, TOSHIAKI IBA

**Affiliations:** 1 Department of Haematology, Manchester University Hospitals, Manchester, UK; 1 Department of Haematology, Manchester University Hospitals, Manchester, UK; 2 Department of Emergency and Disaster Medicine, Juntendo University Graduate School of Medicine, Tokyo, Japan; 2 Department of Emergency and Disaster Medicine, Juntendo University Graduate School of Medicine, Tokyo, Japan

**Keywords:** disseminated intravascular coagulation, coagulopathy, diagnostic criteria, sepsis, anticoagulation

## Abstract

Disseminated intravascular coagulation (DIC) is a common and critical complication in various diseases. There are several diagnostic criteria, such as the International Society on Thrombosis and Haemostasis (ISTH) criteria, the Japanese Society on Thrombosis and Hemostasis (JSTH) criteria, and the Japanese Association for Acute Medicine (JAAM) criteria. Due to the strengths and drawbacks inherent in each diagnostic criterion, it has the potential to cause confusion in clinical settings. It is possible to increase the specificity by making a complex criterion but simple and easy-to-use criteria are demanded in practice. To establish pragmatic criteria using readily available biomarkers, the ISTH focused on DIC arising from sepsis and released sepsis-induced coagulopathy criteria (SIC). A similar approach will aid in constructing a practical diagnostic criterion tailored to each specific background.

Disseminated intravascular coagulation (DIC) was defined as “an acquired syndrome characterized by the intravascular activation of coagulation with loss of localization arising from different causes…” by the Scientific Subcommittee (SSC) on DIC of the International Society on Thrombosis and Haemostasis (ISTH) in 2001^1)^. The essence of this definition was that despite DIC occurring from various backgrounds, it shows a similar hemostatic phenotype which is systemic activation in coagulation that can lead to hemostatic impairment and organ dysfunction. Therefore, the same diagnostic criteria should be applied for the diagnosis regardless of the underlying diseases. This concept has been widely accepted since then, but it is changing along with the significant advances in the management of DIC. For example, anticoagulant therapy using antithrombin or recombinant thrombomodulin was actively initiated at the early stage of DIC in Japan, and the Japanese Association for Acute Medicine (JAAM) established the diagnostic criteria that diagnose the early phase DIC possible^2)^(Table 1). The pathogenic role of disseminated thrombosis was also explored, and it has been accepted that systemic activation in coagulation and inflammation was elicited for the sake of host defense in sepsis^3)^. On the contrary, the temporal hyper-fibrinolysis in trauma-induced coagulopathy has been perceived as the counter-reaction to the massive thrombin generation (thrombin burst) and the effectiveness of anti-fibrinolytic therapy was proven if it was administered at the appropriate time^4)^. Similarly, the understanding of the pathophysiology of coagulation disorder arising from different backgrounds such as Coronavirus disease 2019-associated coagulopathy and heatstroke-induced coagulopathy has been paid attention in these years. Subsequently, an individual diagnostic criterion for each underlying condition was proposed.

**Table 1:** Comparison of ISTH overt DIC, JAAM DIC, and SIC scoring systems

		ISTH overt DIC	JAAM DIC	ISTH SIC
Item	Score	Range	Range	Range
Platelet count ( x10^9^/L)	3	−	< 80 or ≧ 50% decrease within 24h	−
	2	< 50	−	< 100
	1	≧ 50, < 100	120 >, 80 ≦ or ≧ 30% decrease within 24h	≧ 100, < 150
FDP (D-dimer)	3	strong increase	≧ 25 μg/mL (use convert chart)	−
	2	moderate increase	−	−
	1	−	≧ 10, < 25 μg/mL (use convert chart)	−
Prothrombin time	2	≧ 6 sec	−	> 1.4
	1	≧ 3 sec, < 6 sec	≧ 1.2 (PT ratio)	> 1.2, ≦ 1.4 (PT-INR)
Fibrinogen (g/mL)	1	< 100	−	−
SIRS score	1	−	> 3	−
SOFA score	2	−	−	≧2
	1	−	−	1
Total score for DIC or SIC		≧ 5	≧ 4	≧ 4

ISTH, International Society on Thrombosis and Haemostasis; DIC, disseminated intravascular coagulation; JAAM, Japanese Society for Acute Medicine; SIC, Sepsis-induced coagulopathy; SIRS, Systemic Inflammatory Response Syndrome; SOFA, sequential organ failure assessment Total SOFA score is the sum of 4 items (respiratory SOFA, cardiovascular SOFA, hepatic SOFA, and renal SOFA).

Here is a first question. Is it prudent to formulate distinct diagnostic criteria tailored to each background? Along with the advances in pathogenic research, it has become difficult to apply the same diagnostic criteria to all types of DIC. Accordingly, the Japanese Society on Thrombosis and Hemostasis (JSTH) launched a new diagnostic criterion that was divided into three parts depending on the basal conditions; basic type, hematopoietic-disorder type, and infectious type^5)^. In the JSTH criteria, the platelet count was removed for diagnosing the hematopoietic type, and fibrinogen was excluded from the infectious type of DIC. However, these modifications deviate from the original concept of DIC. Besides, the ISTH released new diagnostic criteria which are specifically designed to detect the early phase DIC in sepsis, per se, sepsis-induced coagulopathy (SIC) criteria^6)^. The fault of SIC is the low specificity, requiring the differential diagnosis of the diseases that mimic DIC. On the contrary, the drawbacks of JSTH criteria are the complexity and the costs involved performing molecular markers such as thrombin-antithrombin complex, soluble fibrin, prothrombin fragment_1+2_, and antithrombin activity to make a more accurate diagnosis. Then, the second question arises: whether it is the right direction to make more ‘accurate’ diagnostic criteria.

Prior to delving into the aforementioned arguments, it is necessary to explore the meaning of an 'accurate' diagnosis of DIC. DIC is a conceptual idea, and recent research has elucidated the molecular pathogenic mechanisms of this condition^7)^. Nevertheless, there still exists a wide gap between the pathological findings and the clinical diagnosis based on the laboratory data. Though the sensitive markers, such as molecular markers, endothelial damage markers, microparticles, and damage-associated molecular markers, may be helpful in diagnosing DIC more sensitively, the benefit will be limited in the clinical practice^8)^. We think the ultimate value of diagnostic criteria should be defined from the viewpoint of clinical usefulness. In other words, how much does making a DIC diagnosis impact on the clinical course of the patient is important. For example, the JAAM DIC criteria were designed to determine the timing of anticoagulant therapy^2)^, and its clinical usefulness was shown repeatedly in clinical studies^9, 10)^. Regarding the concept of diagnostic criteria, Gando et al.^11)^ mentioned that they should be composed of readily available markers and easy to use; (2) they should have diagnostic accuracy; and (3) they should display prognostic value, meaning that the diagnostic criteria should be precise and practical.

Currently, there are two options, to make comprehensive or individual, and precise but complex or simple but less accurate, and we still don’t know which way to go. As for simplification, we recently proposed the potential usefulness of the modified version of JAAM DIC diagnostic criteria^12)^ and a simplified version of JSTH DIC diagnostic criteria^13)^. Utmost, what could be the simplest version? In the case of infection-based coagulopathy, we have established the category named ‘SIC’^14)^ using only three items, Sepsis-3 (infection with organ dysfunction)^15)^, platelet count, and prothrombin time. We hope this type of new category can identify the patients with a high risk of death and the cases that benefit from anticoagulant therapy. Certainly, SIC was specifically designed for sepsis-associated DIC and adopted only a minimal number of items, but its performance is considered to be practically sufficient^16)^.

## Funding

No funding was received.

## Author contributions

JT and TI wrote and reviewed the manuscript. Both authors read and approved the final manuscript.

## Conflicts of the interest statement

The authors declare that they have not conflict of interest.
